# Physical Therapy for Traumatic and Non-Traumatic Spinal Cord Injuries in Adults: A Systematic Review

**DOI:** 10.3390/healthcare14142048

**Published:** 2026-07-08

**Authors:** Magdalini Stamou, Nikolaos Manousos, Stylianos Stavrou, Nikolaos Chrysagis, Vasiliki Sakellari

**Affiliations:** Department of Physiotherapy, University of West Attica, 12243 Athens, Greece; phys20683063@uniwa.gr (N.M.); phys20683110@uniwa.gr (S.S.); nchrisagis@uniwa.gr (N.C.); vsakellari@uniwa.gr (V.S.)

**Keywords:** spinal cord injuries, physical therapy modalities, physiotherapy, neurological rehabilitation, adults, traumatic spinal cord injury, non-traumatic spinal cord injury

## Abstract

Background: This systematic review evaluates clinical trials on spinal cord injury (SCI) physical therapy interventions. The objective is to assess the impact of contemporary physical therapy interventions on patients’ functional parameters and recovery outcomes, based on the literature published in the preceding six years. Methods: Inclusion and Exclusion Criteria: Eligible studies included randomized controlled trials (RCTs) and clinical trials involving adult participants with SCI who received physiotherapy interventions. Studies published more than six years ago or those not involving physiotherapy as a primary intervention were excluded. Data Synthesis and Presentation: Results were synthesized qualitatively. Due to substantial clinical and methodological heterogeneity across the included trials, a meta-analysis was not performed, and findings are based entirely on qualitative synthesis. Main outcomes were categorized into primary (muscle strength, walking ability, balance, activities of daily living [ADL]) and secondary outcomes (cardio-respiratory function, mental health, pain management) and are presented in order of the number of supporting studies. Results: Study Numbers and Characteristics: Of 181 records initially identified, 23 studies (all high-quality clinical trials/RCTs; mean PEDro score 7.2) were ultimately included in the review. Main Outcomes and Direction of Effect: The included studies generally reported positive effects of physiotherapy interventions on several functional and physiological outcomes. Statistically significant improvements favoring the physical therapy groups were reported for balance (7 studies), ADLs (4 studies), and respiratory function (6 studies) across the clinical spectrum. Conversely, positive outcomes in muscle strength (12 studies) and gait/walking ability (7 studies) were predominantly localized within motor-incomplete cohorts. Cardiovascular function outcomes (5 studies) remained inconsistent, while secondary psychological metrics and formal mental health parameters (6 studies) showed no statistically significant changes between groups. Discussion: Limitations of the Evidence: The primary limitation identified across the included evidence is the substantial heterogeneity in the specific physical therapy intervention protocols utilized across trials, which limits direct comparison. Interpretation and Implications: Current evidence suggests that tailored physical therapy interventions play an important role in driving functional adaptations and managing specific physiological complications in adult SCI populations. The principal implication of these findings is that, while physical therapy is potentially effective, findings must be interpreted with caution due to protocol diversity, and further high-quality, standardized research is urgently required to establish uniform clinical guidelines. Other: Funding Source: This systematic review received no specific grant or primary funding from any agency in the public, commercial, or not-for-profit sectors.

## 1. Introduction

Spinal cord injury (SCI) represents a profound neurological insult characterized by significant disruption of motor, sensory, and autonomic functions. The underlying pathophysiology follows a complex, biphasic course: an acute phase—marked by immediate mechanical damage and ischaemia—which progresses into a chronic phase involving oxidative stress, a robust inflammatory response, and programmed cell death (apoptosis) [1].

Global estimates indicate an annual incidence of 250,000 to 500,000 new cases worldwide. Demographic data indicate that the majority of patients are male, with a mean age of 37.3 years. Anatomically, the overall distribution of injuries is heavily concentrated in two primary regions: cervical spine injuries account for 51.6% of cases, whereas thoracic spine injuries represent 42.2% [2].

In the European context, incidence rates generally range between 16 and 19.4 per million inhabitants annually; however, most European nations report figures exceeding 20 cases per million, with the exception of the Netherlands, Spain, and Denmark [3]. Fundamentally, SCI is categorized based on the mechanism of injury into traumatic and non-traumatic origins.

Traumatic SCI results from external physical forces. In Europe, the primary causes include road traffic accidents (53%), falls (19%), and sports or recreational activities (18%), followed by other mechanisms such as violence involving firearms or sharp-force trauma [4]. By contrast, non-traumatic SCI arises from internal pathological processes rather than external kinetic forces. Key etiologies include degenerative conditions (e.g., spinal stenosis and osteoarthritis), infectious processes (e.g., viral infections and abscesses), and neoplastic growths [5].

The clinical presentation of SCI is classified according to the severity and permanence of neurological deficits, primarily distinguishing between complete and incomplete injuries, which is standardized through the American Spinal Injury Association (ASIA) Impairment Scale [5]. Within this international standard, the neurological boundaries are defined across four distinct categorical grades: ASIA A (Complete Injury) is characterized by a total absence of motor and sensory function preservation within the lowest sacral segments (S4–S5); ASIA B (Sensory Incomplete) denotes partially preserved sensory function below the neurological level extending through sacral segments S4–S5, while voluntary motor function is entirely absent more than three levels below the lesion on either side; ASIA C (Motor Incomplete) indicates preserved motor function below the neurological level, but more than half of the key muscles present with a muscle grade < 3 (clinically incapable of active movement against gravity); and ASIA D (Motor Incomplete) confirms that motor function is preserved below the neurological level, and at least half or more of the key muscles demonstrate a muscle grade ≥ 3 (capable of active functional movement against gravity) [6]. The severity of the damage further categorizes the resulting disability into paraplegia, defined as injury to the thoracic or upper lumbar spinal cord affecting the trunk, lower limbs, and pelvis with fully preserved upper limb function [7], and tetraplegia, referring to cervical spinal cord damage resulting in impaired function of the upper and lower limbs, as well as the trunk [8].

Patients with spinal cord injuries present with secondary complications affecting multiple physiological systems, including spasticity, osteoporosis, neurogenic bladder, gastrointestinal disorders, respiratory tract infections, neuralgia, pressure ulcers, deep vein thrombosis, hypotension, and hyponatremia [2,9,10,11,12]. Evidence indicates that first-year mortality following spinal cord injury is 8.2% for individuals with tetraplegia (C1–C8, ASIA A–D), compared with 4.1% for those with paraplegia (T1–S5, ASIA A–D) [4,13]. Although traumatic and non-traumatic SCI populations exhibit distinct etiologies, they heavily overlap in these secondary clinical complications and share common physiological mechanisms of deconditioning. Because both cohorts face similar secondary physical challenges and rely on identical pathways of activity-dependent neuroplasticity and muscular reconditioning, evaluating physical therapy interventions concurrently provides a comprehensive, unified understanding of core recovery pathways.

The rehabilitation of patients with SCI necessitates the coordinated engagement of a comprehensive multidisciplinary team [14], within which physical therapy modalities are broadly classified into two primary categories anchored by peer-reviewed evidence: Conventional Physical Therapy, involving targeted therapeutic exercise protocols, passive or active-assisted stretching, and progressive resistance training [15,16,17,18], and Innovative Rehabilitation Methods, utilizing advanced technology such as robotic exoskeletons, virtual reality (VR), EMG-biofeedback, hydrotherapy, functional electrical stimulation (FES), non-invasive mechanical ventilation, and Transcutaneous Electrical Nerve Stimulation (TENS) [19,20,21,22,23,24]. While these modalities have expanded over the preceding six years, current clinical evidence remains fragmented across highly heterogeneous intervention durations and training intensities. To address this knowledge gap and map the clinical direction of effects, the formal objective of this systematic review was to evaluate the literature from the preceding six years (2019–2025), answering the core review question framed via the PICO structure at the absolute end of this introduction:

Population (P): Adult participants (≥19 years) diagnosed with either traumatic or non-traumatic spinal cord injury.
Intervention (I): Physical therapy or physiotherapy-based interventions (conventional or innovative technology-assisted modalities).
  Comparison (C): Conventional care, alternative physical exercises, sham interventions, or passive control conditions.
Outcomes (O): Primary outcomes of muscle strength, walking ability, balance, and activities of daily living (ADL), and secondary outcomes of cardiorespiratory function, pain management, and mental health parameters.

## 2. Materials and Methods

This systematic review was conducted in strict accordance with the PRISMA (Preferred Reporting Items for Systematic Reviews and Meta-analyses) guidelines.

### 2.1. Inclusion and Exclusion Criteria

For inclusion, studies were required to meet the following criteria: (1) randomized controlled trials (RCTs) and clinical trials; (2) published in English; (3) published within a six-year window (1 January 2019 to 30 April 2025); and (4) involving adult participants (aged ≥ 19 years) diagnosed with either traumatic or non-traumatic spinal cord injury. The primary outcomes evaluated were the effects of the interventions on muscle strength, gait, balance, and activities of daily living (ADLs). Secondary outcomes of interest encompassed respiratory and cardiovascular function, pain management, and mental health parameters.

Studies were excluded if they involved: (1) individuals without a stable or safe health status that would clinically contraindicate physical therapy interventions; (2) co-existing neurological or medical conditions other than SCI, such as ischemic or hemorrhagic stroke, malignancies, or isolated radiculopathies; (3) individuals who had sustained any other major musculoskeletal or neurological injury within 6 months prior to the initiation of the intervention. Additionally, as specified in our pre-registered protocol, primary studies with a PEDro score below 6/10 were excluded to ensure the quality of the evidence synthesis. Regarding the multidisciplinary nature of spinal cord injury rehabilitation, interventions were eligible if physiotherapy (conventional exercises or technology-assisted physical training) was investigated as the primary independent variable, with outcomes evaluating physical or functional parameters. Articles were excluded if the intervention was not physiotherapy-based or if outcomes were confounded by the concurrent involvement of other medical specialties (such as surgical procedures, pharmaceutical treatments, or cell transplantation) where the independent effect of the physical therapy modality could not be isolated.

### 2.2. Information Sources and Search Strategy

Comprehensive literature searches were conducted in April 2025 across three electronic databases: PubMed (searched on 5 April 2025), PEDro (searched on 5 April 2025), and Scopus (searched on 12 April 2025). The search strategy utilized a comprehensive combination of Medical Subject Headings (MeSH) terms, free-text keywords, Boolean operators (AND/OR), field tags, and specific database filters related to the clinical condition and the nature of the physical intervention.

The detailed electronic search strategies and exact syntaxes applied by database were executed as follows:

PubMed (Searched on 5 April 2025): The exact search string executed was: (“Spinal Cord Injuries”[Mesh] OR “Spinal Cord Injury”[All Fields] OR “SCI”[All Fields] OR “Paraplegia”[All Fields] OR “Tetraplegia”[All Fields]) AND (“Physiotherapy”[All Fields] OR “Physical Therapy”[All Fields] OR “Therapeutic Exercise”[All Fields] OR “Exercise”[Mesh] OR “Exercise”[All Fields]) AND (“Rehabilitation”[Mesh] OR “Rehabilitation”[All Fields] OR “Intervention”[All Fields]). The filters applied restricted the results to articles published in the English language between 1 January 2019, and 5 April 2025, involving adult participants aged 19 years and older. This search strategy retrieved a total of 70 records.

Scopus (Searched on 12 April 2025): The search strategy was adapted for Scopus syntax using the following exact string: (TITLE-ABS-KEY (“Spinal Cord Injur*” OR “SCI” OR “Paraplegia” OR “Tetraplegia”) AND TITLE-ABS-KEY (“Physiotherap*” OR “Physical Therapy” OR “Therapeutic Exercise”) AND TITLE-ABS-KEY (“Rehabilitation” OR “Intervention”)) AND PUBYEAR > 2018 AND PUBYEAR < 2026 AND (LIMIT-TO (LANGUAGE, “English”)). The search was limited to English-language literature published within the 2019–2025 eligibility period. This query yielded a total of 106 records.

PEDro (Searched on 5 April 2025): Due to the specialized indexing architecture of the Physiotherapy Evidence Database, the advanced search function was utilized with targeted field restrictions. The search term “Spinal Cord Injury” was executed within the Abstract & Title field, while the Method field was restricted to “clinical trial” via the database’s standardized dropdown menu. The Published Since filter was set to 2019 to capture trials within the 2019–2025 eligibility window. Because PEDro exclusively indexes the physical therapy literature, broader terms like “physiotherapy” or multi-layered therapy sub-filters were omitted to prevent excessive over-filtering of relevant evidence. This targeted advanced search strategy successfully retrieved a total of 5 records.

Across all three electronic databases, the pooled initial literature search retrieved a cumulative total of 181 records.

### 2.3. Study Selection and PRISMA Flow Diagram

**Figure 1:**
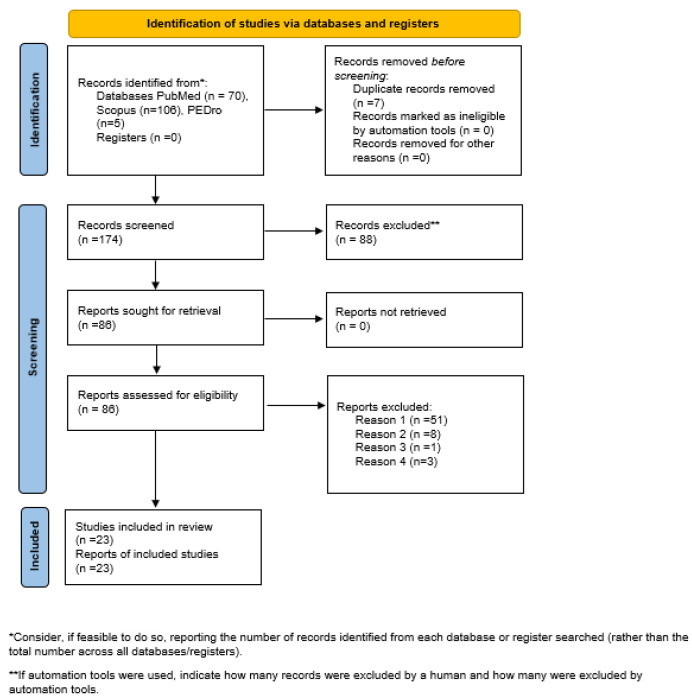
Source: Page MJ et al. BMJ 2021; 372:n71. doi: 10.1136/bmj.n71 [25].

The systematic review protocol was pre-registered on the Open Science Framework (OSF) platform as part of an ongoing institutional research project and assigned DOI: 10.17605/OSF.IO/TPQ9S (accessible at https://osf.io/v64m2, accessed on 13 May 2026). No amendments or modifications were made to the protocol following its initial registration. To comply fully with PRISMA reporting standards, the study selection process is systematically documented via a formal PRISMA flow diagram integrated within the manuscript (Figure 1).

The screening workflow progressed with absolute numerical consistency across the following sequential phases:

Identification: A total of 181 records were retrieved from the initial electronic database searches (PubMed: 70, Scopus: 106, PEDro: 5). No additional records were identified through citation chasing or external gray literature sources.

Duplicate Removal: Out of the 181 compiled records, exactly 7 duplicate articles were identified and electronically removed using the Rayyan web platform, leaving 174 unique records for secondary screening.

Title and Abstract Screening: Two independent researchers (N.M. and S.S.) screened the titles and abstracts of the 174 unique records against the pre-specified inclusion/exclusion criteria. This phase resulted in the exclusion of 88 records due to non-compliance with population age, non-physiotherapy interventions, or ineligible study designs.

Full-Text Eligibility Assessment: The remaining 86 records were retrieved and assessed for eligibility. Following independent review by two screeners (N.M. and S.S.), 63 full-text articles were excluded with documented justifications: 51 due to a PEDro score below 6/10, 8 due to the absence of a dedicated physical therapy intervention, 1 due to an inappropriate patient population, and 3 because the full text was irretrievable. Discrepancies were resolved through discussion with a third reviewer (M.S.).

Inclusion: A final total of 23 high-quality clinical trials/RCTs met all methodological requirements and were included in the qualitative narrative synthesis.

### 2.4. Data Extraction and Synthesis

Data extraction was conducted systematically using standardized matrices. The collated data points included the number of participants, specific characteristics of the experimental and control group interventions, assessment scales, clinical outcome tools, and the primary results reported in each study to enable cross-trial comparison. Due to substantial clinical, neurological, and methodological heterogeneity across the included protocols—specifically variations in lesion completeness (complete vs. incomplete), injury levels (cervical vs. thoracic), and physical therapy intervention archetypes (robotic, FES, aquatic, or conventional training)—a quantitative meta-analysis was not viable, and findings were synthesized via a qualitative narrative approach.

### 2.5. Quality and Bias Assessment

The methodological quality and risk of bias of the included trials were independently evaluated by two researchers using the PEDro scale.

Sensitivity Analysis: A qualitative sensitivity analysis was pre-planned to assess the robustness of the synthesized findings by evaluating whether overall conclusions would change if studies with lower methodological quality (PEDro score < 6/10) were excluded from the narrative synthesis. Since all 23 included trials maintained moderate-to-high methodological quality (with a mean PEDro score of 7.2 and no individual study scoring below 6/10), the consistency of positive outcomes across evaluated domains confirmed the robustness of the primary findings.

Reporting Bias Assessment: Risk of bias due to missing results (reporting bias) was qualitatively evaluated by assessing whether the included studies reported findings for all targeted clinical parameters, as well as through specific items on the PEDro scale pertaining to investigator/participant blinding and key outcome reporting.

### 2.6. Clinical Outcome Measures and Psychometric Validation

To ensure high methodological transparency, reproducibility, and clinical interpretability, the outcome measures evaluated across the included traumatic and non-traumatic SCI trials were classified into six distinct clinical domains. Every utilized evaluation tool was mapped against its established psychometric validation properties, explicitly anchored to its documented test–retest reliability indices (Intraclass Correlation Coefficients, ICC) and construct validity parameters for spinal lesion cohorts:

1. Muscle Strength and Neurological Metrics: Motor recovery and baseline neurological tracking were primary metrics across trials, assessed via the International Standards for Neurological Classification of Spinal Cord Injury (ISNCSCI) motor score and manual muscle testing (MMT) using the Medical Research Council (MRC) scale. These core tools demonstrate excellent inter-rater reliability (ICC ≥ 0.92) and strong construct validity when tracking progressive neurological adaptations in motor-incomplete and complete spinal lesions [26,27].
2. Gait, Ambulation, and Spasticity: Spatiotemporal gait parameters, functional walking velocity, and spasticity changes were systematically captured using the Walking Index for Spinal Cord Injury II (WISCI-II), the 10-Meter Walk Test (10MWT), the 6-Minute Walk Test (6MWT), and the Modified Ashworth Scale (MAS). The 10MWT and 6MWT exhibit exceptional test–retest reliability (ICC = 0.96–0.98) and high responsiveness to changes in ambulatory capacity, while the WISCI-II provides precise construct validity for structural bracing and physical assistance dependencies [28,29,30].
3. Postural Control and Balance: Dynamic and static balance adaptations during seated or standing configurations were predominantly mapped using the Berg Balance Scale (BBS), the Activities-specific Balance Confidence (ABC) scale, and the Timed Up and Go (TUG) test. These metrics show robust psychometric stability (ICC ≥ 0.88), serving as validated predictors of fall risks and mechanical stability zones during complex physical therapy movements [31,32,33].
4. Activities of Daily Living (ADL) and Autonomy: Functional independence and self-care autonomy changes were quantified using the Spinal Cord Independence Measure III (SCIM-III) and the Functional Independence Measure (FIM). The SCIM-III is a highly specific, globally validated tool for spinal cord pathologies, showing superior sensitivity and construct validity compared to generic scales, with a documented inter-rater reliability ICC ≥ 0.94 [34,35].
5. Cardiorespiratory and Endurance Parameters: Autonomic, circulatory, and respiratory adaptations resulting from targeted exercise loading were evaluated via peak oxygen uptake (VO2 peak), forced expiratory volume in 1 s (FEV_1_), forced vital capacity (FVC), and peak expiratory flow (PEF). These physiological metrics demonstrate strong test–retest reproducibility (ICC = 0.85–0.91) and are validated indicators of respiratory muscle reconditioning and cardiovascular endurance capacity [36,37,38,39].
6. Quality of Life (QoL), Mental Health, and Pain Assessment: Secondary subjective outcomes were tracked using the Visual Analog Scale (VAS) for pain intensity, the Spinal Cord Injury Secondary Conditions Scale (SCI-SCS), the 36-Item Short Form Health Survey (SF-36), and the Hospital Anxiety and Depression Scale (HADS). These tools demonstrate good internal consistency (Cronbach’s alpha ≥ 0.81) and structural validity for capturing the broader biopsychosocial impacts of rehabilitation protocols [40,41,42,43,44,45].

## 3. Results

### 3.1. Study Selection Flow

The study selection process is systematically detailed in the PRISMA Flow Diagram (Figure 1). A total of 181 records were initially identified through comprehensive database searching, including 70 from PubMed, 5 from PEDro, and 106 from Scopus. These citations were imported into Rayyan, where 7 duplicate records were identified and subsequently removed.

Following the initial screening of titles and abstracts of the remaining 174 unique records, 88 articles were excluded. Consequently, 86 reports were sought for retrieval. At this stage, 63 records were excluded based on predefined criteria: moderate-to-low methodological quality with a PEDro score below 6/10 (*n* = 51), absence of a dedicated physical therapy intervention (*n* = 8), an inappropriate target patient population (*n* = 1), and irretrievable full text (*n* = 3). Ultimately, exactly 23 clinical trials fulfilled all strict eligibility requirements and were progressed to the final narrative synthesis.

To provide a comprehensive overview of the trial characteristics and the broader clinical outcomes of the included evidence, additional analytical details are provided in the Supplementary Materials. Specifically, Supplementary Table S1 presents a categorical matrix illustrating the structural distribution and therapeutic efficacy of the included trials across primary functional evaluation domains. Furthermore, the detailed demographic and clinical stratification of the total pooled participant sample (*N* = 730) is mapped in Supplementary Table S2, highlighting the distributions across injury etiologies, neurological levels, and chronicity phases.

### 3.2. Participant Characteristics

To provide a structured overview of the current literature, the synthesized data were organized into separate analytical domains. Table 1 outlines the specific injury types, clinical pathologies, and sample sizes for each included trial. As shown, the clinical spectrum spans a wide range of neurological completeness and injury levels across both experimental and control groups.

To elucidate the specific active mechanisms under investigation, Table 2 provides a highly structured breakdown of the physical therapy intervention characteristics. This includes precise descriptions of the specialized modalities assigned to the experimental arms (such as robotic exoskeletons, virtual reality environments, and neurostimulation setups), directly contrasted against their respective control conditions.

To ensure methodological transparency and validate the reliability of the evidence, Table 3 indexes the specific standardized testing batteries, questionnaires, and assessment instruments deployed across the clinical spectrum. These validated psychometric tracking tools are categorized alongside their primary clinical targets and the targeted directions of their clinical effects.

Finally, Table 4 establishes a rigorous qualitative synthesis of the primary findings. Addressing a vital clinical parameter, this table explicitly highlights the observed directions of therapeutic effect; documents their corresponding levels of statistical significance; and contextualizes them across varying short-, medium-, or long-term post-baseline follow-up windows.

### 3.3. Baseline Characteristics of Participants

To ground the narrative synthesis in precise quantitative terms, the cumulative, detailed demographic data of the pooled sample are broken down in Table 5. This structure accounts for missing data parameters explicitly, allowing clinical classification values to be accurately presented across both intervention and control arms.

Regarding the pooled demographic totals presented in Table 5, missing data from individual studies were handled by calculating cumulative sums based exclusively on reporting cohorts. Specifically, the total sample consists of 733 participants; however, cumulative gender metrics are reflective of the trials that explicitly documented sex distribution, and injury clinical classifications represent the subset of trials with complete reporting. Missing data were not imputed, and percentages are adjusted based on the available denominators to ensure epidemiological accuracy.

A dedicated sub-analysis was performed to map the representation of injury etiologies across the pooled sample. Out of the 733 participants, 647 individuals belonged to trials that provided explicit data regarding traumatic vs. non-traumatic onset. Within these reporting cohorts, a cumulative total of 546 patients sustained a traumatic spinal cord injury (tSCI; 274 in the Intervention Group and 272 in the Control Group). Conversely, 101 patients presented with non-traumatic spinal cord injury (ntSCI; 55 in the Intervention Group and 46 in the Control Group), primarily localized within 8 mixed-cohort trials that quantified pathological or medical underlying mechanisms. As detailed in Table 5, the overall male-to-female ratio across the reporting studies was approximately 3:1. Regarding the anatomical level of injury, a total of 418 participants with tetraplegia (injury level: C1–C8) and 315 participants with paraplegia (injury level: T1 and below) were recorded. Participants across all included studies were systematically evaluated using the American Spinal Injury Association (ASIA) Impairment Scale (AIS). Based on this standardized classification, ASIA A indicates complete neurological impairment, whereas ASIA B–D denote incomplete impairment. Based on the subset of trials providing explicit neurological grading data, 312 patients with complete lesions and 415 patients with incomplete lesions were identified across the pooled sample.

### 3.4. Analysis of Heterogeneity

Synthesis of the included studies revealed substantial clinical and methodological heterogeneity, which directly influenced the comparability of functional outcomes and precluded the statistical pooling of data. These variations were categorized across the following domains:

Patient Stratification (Clinical Heterogeneity): The pooled study sample spanned a broad neurological spectrum, encompassing both tetraplegia (C1–C8) and paraplegia (T1–S5). The concurrent inclusion of both complete or near-complete (ASIA A–B) and incomplete (ASIA C–D) spinal cord injuries introduced inherent variability in the baseline motor and sensory potential of the participants, thereby limiting the uniformity of the interventions’ therapeutic impact. Specifically, statistically significant improvements in functional overground gait velocity were isolated within motor-incomplete (ASIA C and D) subacute cohorts of the sample, where anatomical sparing of descending spinal tracts facilitates locomotor recovery. Conversely, localized neurophysiological muscular adaptations and electromyographic activity changes were observed to extend into sensory-incomplete but motor-complete profiles (ASIA B), indicating that targeted technological interventions can elicit subclinical neuromuscular responses even in the absence of functional voluntary movement. At the same time, functional adaptations in dynamic postural balance and respiratory capacity (specifically maximum inspiratory pressure, PImax) were observed across both neurologically complete (ASIA A) and incomplete cohorts within the pooled sample, demonstrating that targeted training can drive functional gains regardless of baseline sensory or motor completeness through the recruitment of neurocompensatory upper-truncal mechanisms. Furthermore, variations in chronicity (subacute versus chronic lesions) and etiology (traumatic versus non-traumatic spinal cord injuries) added further layers of clinical diversity.

Intervention Protocols and Methodological Heterogeneity: Substantial divergence was observed in the structure of physical therapy programmes across the following domains:

Duration: The active intervention periods across the included trials were primarily short-to-medium term, typically ranging from 2 to 12 weeks. However, a notable source of methodological variation was introduced by a single multicenter pragmatic trial [46], which featured a highly variable long-term duration span tracking participants from 1.5 up to 20 months.

Dosage, Frequency, and Intensity: Training protocols varied widely, from low-intensity therapeutic exercises to moderate-to-high-intensity programmes employing progressive resistance, functional electrical stimulation, or advanced mechanical loading.

Control Conditions: Comparison groups differed extensively, ranging from standard flat-surface overground walking and passive mobilization to educational-only control conditions or sham stimulation setups.

Assessment Timelines and Outcome Measures: Assessment time points were not synchronized across trials. Short-to-medium term post-intervention evaluations were frequently conducted at 2, 4, 6, 8, or 12 weeks post-baseline, whereas a small subset of trials extended final longitudinal measurements to 16 weeks or 6 months, alongside the highly variable chronic timeline tracked by [46]. Across these timelines, studies utilized over 40 distinct psychometric scales and physiological metrics.

Due to this compounding clinical and methodological heterogeneity—where pooling data would lead to severely confounded and clinically uninterpretable summary estimates—a quantitative meta-analysis was deemed inappropriate. Consequently, a rigorous qualitative narrative synthesis was executed as the mathematically and clinically sound alternative, a decision explicitly outlined in both the Methods and Results.

### 3.5. Methodological Quality and Risk of Bias Assessment

The methodological quality and risk of bias of the included studies were evaluated using the PEDro scale. All trials were independently scored on a 0–10 scale by two researchers (N.M. and S.S.) (Table 6). Scores ranged from 6/10 to 10/10, yielding a mean of 7.2. While this average indicates a generally solid evidence base, trials scoring 6/10 must be appropriately classified as presenting moderate rather than high methodological quality, requiring a more cautious and conservative interpretation of their reported trends.

A detailed analysis of the individual PEDro items highlighted several common methodological limitations across the included trials. The most pervasive weakness was the systematic lack of participant and therapist blinding, an inherent challenge in physical therapy interventions where active cooperation is mandatory. Additionally, several lower-scoring studies (6/10) were characterized by small sample sizes, unclear allocation concealment protocols, and an absence of a formal intention-to-treat analysis. These structural weaknesses underscore the need for a cautious evaluation of the magnitude of effect sizes reported in the primary literature.

### 3.6. Methodological Quality Influence on Evidence Weight

The overall robustness of the synthesized findings was evaluated by contextualizing the direction of effects against the methodological quality of the primary evidence. Out of the 23 included trials, 8 achieved high methodological quality (PEDro scores 8–10/10), while 15 presented moderate quality (PEDro scores 6–7/10). When focusing on the highest-quality trials (scores ≥ 8/10), the positive therapeutic direction on core recovery pathways—specifically in respiratory muscle reconditioning and localized daily living autonomy (ADLs)—remained consistently supported. This cross-comparison indicates that the broader non-significant trends observed in mental health and cardiovascular parameters were a reflection of genuine clinical variations rather than a systemic distortion caused by lower-scoring primary studies.

### 3.7. Reporting Biases

The qualitative assessment of the potential bias due to missing results indicated a low risk of reporting bias across all synthesized outcome domains. The included trials demonstrated high internal consistency between pre-specified protocol parameters and published datasets, with no explicit evidence of selective outcome reporting. Standardized PEDro scale item evaluation further confirmed adequate completeness of longitudinal follow-up and thorough reporting of key post-intervention metrics, suggesting that the synthesized results were not systematically distorted by missing data channels.

The primary outcomes targeted the efficacy of targeted physiotherapy interventions with respect to muscle strength, gait parameters, balance control, and activities of daily living (ADLs). Secondary outcomes encompassed respiratory mechanics, cardiovascular profiles, pain intensity indices, and specific mental health parameters—including anxiety, depression, and sleep quality. To maximize scannability and structure the evidence weight as requested, the evaluation instruments deployed across the selected literature are systematically categorized within Table 7.

### 3.8. Muscle Strength Outcomes

Assessment of localized muscular adaptations, isometric contractions, and anaerobic reconditioning was conducted across 12 trials, combining a cumulative sample of 328 participants. Methodological tools included the Lower Extremity Motor Score (LEMS), the Five-Times-Sit-to-Stand (FTSTS) test, stepping power metrics, Maximal Voluntary Contraction (MVC), Peak Torque (PT), and Peak Power Output (PPO). Synthesized evidence demonstrates that statistically significant and clinically meaningful strength adaptations were heavily moderated by baseline lesion completeness and intervention specificities.

Lower Extremity Motor Score (LEMS): Evaluation of lower limb reconditioning via the LEMS spanned 5 trials [19,20,21,22,23]. Statistically significant and clinically meaningful improvements were exclusively isolated within motor-incomplete subacute cohorts, where body-weight-supported gait re-training guided by interactive biofeedback induced superior score increments ranging from 3.6 [50] to 4.8 points [21] in the active intervention groups. Conversely, chronic or neurologically complete spinal cord injury cohorts yielded non-significant findings (*p* > 0.05), presenting negligible intra-group variations between −1 [49] and 1 point [20,21], thereby validating that structural spinal sparing weights the ultimate magnitude of motor adaptations.

Five-Times-Sit-to-Stand Test (FTSTS): Dynamic lower limb functional power was monitored across 3 studies [19,24,47]. A distinct positive trend in reducing task completion times was reported at short-term follow-up (2 to 4 weeks), with progressive overground walking training on varied surfaces [47] and dual-task obstacle crossing [60] triggering significant time reductions between −1.33 [60] and −3.75 s [47]. However, these achievements demonstrated limited long-term sustainability; prospective medium-term re-evaluation at 6 months revealed a notable performance regression (+0.92 s) in the active training arm [47].

Maximal Voluntary Contraction (MVC) and Strength: Upper and lower limb volumetric muscular capacity was tracked across 3 trials [22,52,54]. Targeted physical reconditioning achieved highly significant and clinically meaningful baseline-to-final strength gains. For upper limb structures, experimental protocols elicited voluntary strength expansions of 40 to 50 Nm compared to stable control parameters [54]. For lower limb reconditioning, active groups yielded a substantial increase of 38.6 Nm, contrasting sharply with negligible control group variations (0.5 Nm) [50]. At a short-term timeline of 8 weeks, intensive voluntary contraction protocols further established a significant 3.3-point strength improvement [52].

Anaerobic and Power Output Metrics (Stepping Power, PT, PPO): Anaerobic performance parameters were synthesized across 4 distinct configurations [19,49,57,58]. Task-specific seated strength exercises triggered a highly significant intra-group increase of 31 Watts (*p* < 0.01) in stepping power for non-ambulatory cohorts, while ambulatory counter-protocols remained unchanged [49]. Upper limb Peak Torque analysis confirmed consistent, statistically significant intervention margins of 5–10 Nm across all tested mechanical angles (0° to 180°) [22]. Finally, maximum anaerobic Peak Power Output (PPO) demonstrated definitive between-group statistical superiority favoring active groups, with post-intervention increments reaching up to 20 Watts [58] or showing a 4 Watts net difference [57].

### 3.9. Gait Ability Outcomes

Walking capacity and spatiotemporal ambulatory parameters were synthesized across 7 studies, combining a cumulative sample of 218 participants [19,20,22,23,24,46,47]. Assessment metrics integrated a range of subjective, functional, and technological tracking devices, including the Patient-Reported Outcomes Measurement Information System (PROMIS), the 10-Meter Walk Test (10MWT), the Walking Index for Spinal Cord Injury (WISCI-II), the Timed Up and Go (TUG) test, and the Spinal Cord Injury Functional Ambulation Profile (SCI-FAP).

Subjective and Functional Baselines (PROMIS, SCI-FAP): Qualitative synthesis of the broader behavioral parameters demonstrated entirely non-significant between-group differences (*p* > 0.05). Both the self-reported, patient-centric PROMIS scale [19,49] and the task-oriented, objective SCI-FAP profiling battery [46] failed to establish clinical superiority for innovative experimental models over established conventional treatment channels.

Gait Velocity Adaptations (10MWT): Long-term and short-term trends regarding overground gait speed adjustments remained clinically modest and highly fragmented. At a short-term timeline of 4 weeks, progressive surface-varied training stimulated a significant velocity expansion of 0.13 m/s, which completely deteriorated back to near-baseline values during medium-term follow-up at 6 months [47]. Conversely, conventional training counter-groups demonstrated slower but steady longitudinal progress, establishing a clinically meaningful long-term cumulative speed expansion of 0.4 m/s [47]. Across remaining trials, overground speed changes were statistically equivalent between groups, rarely exceeding minor velocity increments of 0.1 m/s [20,24].

Ambulatory Independence and Assistive Device Reductions (WISCI-II): Technology-driven locomotor retraining generated highly localized, statistically significant benefits. Robotic-assisted body-weight-supported treadmill training achieved a statistically superior and clinically relevant improvement of 1.7 points on the WISCI-II scale, whereas passive lower-limb mobilization control paths triggered a negligible adaptation of 0.1 points [50]. However, when directly contrasting two distinct active intervention paradigms against each other (e.g., robotic versus aquatic strategies), no statistically significant differences were captured [21].

Dynamic Balance and Complex Obstacle Performance (TUG): The most substantial and definitive therapeutic gains across the walking spectrum were clustered within the TUG data. Intensive loco-motor retraining on varied, unstable overground configurations provoked a massive, highly significant short-term reduction of −7.42 s in overall execution time, dramatically outperforming flat-surface training, which yielded a minor time drop of −1.49 s [47]. Furthermore, systematically expanding environmental complexity and raising kinetic obstacle thresholds successfully induced task-specific, short-term functional response time adaptations [60].

### 3.10. Postural Control and Balance Outcomes

The impact of specialized physical therapy interventions on postural control and balance was evaluated across 7 studies pooling a cumulative sample of 200 participants [19,20,24,46,47,50,51]. Methodological tracking involved 6 standardized clinical tools: the Modified Functional Reach (MFR) test, the Berg Balance Scale (BBS), the Activities-specific Balance Confidence (ABC) scale, the Function in Sitting Test (FIST), the Quadruped Unilateral Reaching test, and the Tall-Kneeling test. Collectively, the synthesized findings highlight a statistically significant (*p* < 0.05) and clinically meaningful effectiveness of technology-integrated and task-specific protocols over conventional therapeutic approaches.

Sitting Balance and Postural Control Adaptations: At short-term follow-up timelines, interventions incorporating immersive Virtual Reality (VR) and specialized neuromuscular activation demonstrated absolute superiority regarding sitting stability and trunk control. Immersive VR-based training induced significantly higher short-term gains in active sitting balance compared to electrical stimulation (ES) protocols alone, driving distinct statistical superiority across both objective MFR and FIST scores [23]. Similarly, targeted core exercise pathways, enhanced with or without concurrent ES, triggered significant structural advancements in postural stability during challenging configurations, such as quadruped reaching and tall-kneeling milestones [51]. In sharp contrast, traditional overground physical therapy paths generated negligible functional progress or triggered a measurable post-intervention decline in trunk performance metrics [51].

Functional Standing Balance and Mobility Confidence: Clinical evaluation of dynamic overground reconditioning versus non-ambulatory channels established a clear, positive direction of effect. Specialized ambulatory (walking-based) loco-motor protocols were markedly more effective than non-ambulatory variants for driving re-education of clinical balance (BBS) and balance-related self-efficacy metrics (ABC scale) [49]. Specifically, the ABC scale tracking confirmed a highly significant post-intervention improvement favoring the ambulatory configurations (*p* < 0.01) [49].

Sustainability and Conventional Comparators: Therapeutic outcomes associated with standard, non-specific physical therapy regimens remained highly inconsistent and non-significant across the compiled evidence. While specialized body-weight-supported gait training demonstrated a modest short-term advantage over conventional methods by mitigating longitudinal declines in raw MFR scores [50], alternative testing showed that standard, traditional rehabilitation protocols paradoxically achieved a higher cumulative long-term increase in balance-related confidence (ABC scale) when directly contrasted against structured, pragmatic walking adaptability training [46]. Notably, across these balance dimensions, neurocompensatory adaptations were consistently documented across the full clinical spectrum, yielding functional progress regardless of baseline sensory or motor completeness.

### 3.11. Activities of Daily Living (ADLs) Outcomes

Functional independence and self-care autonomy were quantified across 4 clinical trials encompassing a cumulative sample of 86 participants [22,50,51,60]. Evaluation methodologies utilized the Spinal Cord Independence Measure (SCIM-III) and the Modified Barthel Index (MBI). Synthesized evidence demonstrates a robust, positive trend, with 3 out of the 4 included studies achieving distinct statistical significance and clinically meaningful functional gains favoring the active rehabilitation or technology-assisted arms.

Independence and Autonomy Adaptations (SCIM-III): Targeted exercise loading and interactive digital platforms yielded highly significant short-term improvements in functional outcomes. At a short-term timeline of 4 weeks, immersive Virtual Reality (VR) frameworks and standalone electrical stimulation paths induced massive, statistically significant gains of 15 and 18 SCIM-III points, respectively [23]. Similarly, task-specific reconditioning demonstrated absolute clinical superiority over conventional designs; progressive weight-bearing mat exercises integrated with concurrent Functional Electrical Stimulation (FES) prompted an improvement of 3.8 points, and standalone exercises triggered a 2.4-point increase, whereas standard physiotherapy control groups resulted in a minor functional decline of −0.3 points [51]. Furthermore, advanced robotic-assisted body-weight-supported treadmill training combined with lower-limb EMG biofeedback established statistically significant, long-term enhancements in global functional independence and overground gait autonomy [50].

Self-Care Re-education Metrics (MBI): Specialized neuromuscular feedback setups proved highly effective for driving everyday clinical autonomy. Tracking via the MBI at final post-intervention timelines confirmed that targeted EMG biofeedback protocols generated a substantial, statistically significant cumulative score increase of 8.53 points [60]. Conversely, conventional physical therapy control paths achieved less than half of that progression, yielding a modest and clinically restricted expansion of 4.37 points [60]. Collectively, these quantified improvements across complete and incomplete injury profiles translated directly into optimized micro-environmental autonomy, enabling individuals to perform transfers and daily self-care tasks with significantly reduced caregiver dependence.

### 3.12. Respiratory Function Outcomes

Pulmonary reconditioning and respiratory mechanics were investigated across 6 clinical trials, combining a cumulative sample of 266 participants [21,22,54,55,56,57]. The qualitative synthesis establishes that target-specific physical therapy protocols drive highly effective, statistically significant (*p* < 0.05) physiological adaptations across 5 out of the 6 analyzed papers [21,22,54,55,56,57].

Targeted Respiratory Muscle Training (RMT): Specialized RMT employing pressure-threshold resistance devices elicited substantial, long-term increments in ventilatory reconditioning [55,56]. Active RMT protocols triggered a statistically significant and clinically relevant expansion in Maximal Inspiratory Pressure (PImax) and Maximal Expiratory Pressure (PEmax) across all evaluated lesion profiles, alongside significant enhancements in Forced Vital Capacity (FVC) [56]. Conversely, sham training arms failed to induce physiological progress and presented a significantly higher incidence of secondary pulmonary complications [56].

Robotic and Mechanical Feedback Environments: Advanced technological interventions exerted a powerful systemic crossover effect on respiratory parameters [21,22]. Overground exoskeleton walking and robotic-assisted body-weight-supported treadmill training integrated with interactive biofeedback significantly optimized pulmonary flow-volume indices, triggering distinct advancements in Peak Expiratory Flow (PEF) and Forced Expiratory Volume in 1 s (FEV_1_) [21,22].

Comprehensive Pulmonary Programs and Well-being: Specialised pulmonary rehabilitation setups and threshold inspiratory training protocols demonstrated the highest clinical efficacy for optimizing patient-reported metrics [55,57]. Active groups achieved significantly higher scores across formal wheelchair aerobic, shuttle run, and propulsion testing batteries, while concurrently documenting a statistically meaningful reduction in exertion dyspnea and sustained improvements in respiratory-related quality of life [55,57]. Notably, these objective physiological and volumetric lung adaptations were observed consistently across the clinical spectrum, with both neurologically complete and incomplete spinal cord injury cohorts exhibiting comparable respiratory advancements [21,56].

### 3.13. Cardiovascular Function Outcomes

Cardiovascular performance, metabolic markers, and autonomic hemodynamic regulation were evaluated across 5 studies pooling a cumulative sample of 131 participants [22,23,48,57,58]. Methodological evaluations tracked a diverse array of indices, including peak oxygen consumption (VO2peak), 6-Minute Walk Test (6MWT) distances, resting systolic and diastolic blood pressure, heart rate variability, and submaximal aerobic endurance metrics. Synthesized evidence indicates that statistically significant improvements in cardiovascular parameters were highly inconsistent across the reviewed literature, with measurable clinical gains which were moderated by intervention type and intensity.

Task-Specific Training and FES Integration: High-intensity whole-body exercise integrated with Functional Electrical Stimulation (FES) and targeted task-specific retraining yielded the most robust and statistically significant enhancements in central cardiovascular fitness. Hybrid FES-assisted rowing [57] and intensive overground ambulatory interventions [48] achieved superior, clinically meaningful gains in maximal aerobic capacity (VO2max) and walking distances compared to conventional passive rehabilitation programs. Furthermore, whole-body hybrid FES loading demonstrated definitive therapeutic efficacy in blood pressure regulation, triggering a significantly greater increase in cardiovagal baroreflex sensitivity among participants with paraplegia than those with tetraplegia [48].

Respiratory Muscle Training Crossover Effects: Targeted respiratory rehabilitation demonstrated a substantial, statistically significant secondary crossover effect on cardiovascular endurance. Manual wheelchair users undergoing structured pressure-threshold conditioning protocols achieved indirect yet meaningful advancements in central circulatory drive, which translated to optimized physical propulsion performance and enhanced upper-limb aerobic threshold capacities.

Robotic Modalities versus Active Aerobic Pathways: Although advanced robotic systems and powered exoskeletons safely stimulated submaximal mechanical parameters, they yielded non-significant or inferior results regarding active oxygen uptake efficiency when contrasted directly against fluid hydrotherapy [21] or specialized overground aerobic training. Highly intensive interval protocols (HIIT) successfully elicited significantly higher acute cardiovascular responses than moderate-intensity continuous pathways, though final peak power output improvements remained statistically equivalent between the active cohorts (*p* > 0.05) [58]. Notably, these specialized metabolic and hemodynamic adaptations were observed consistently across both complete and incomplete spinal lesion profiles, confirming that targeted cardiac loading can drive systemic adaptations regardless of baseline sensory or motor completeness.

### 3.14. Mental Health Outcomes

Interventions focusing on holistic rehabilitation, specialized behavioral conditioning, and high-intensity exercise loading were evaluated across 6 studies pooling a cumulative sample of 238 participants [20,22,53,58,59,62]. Although overall physical retraining drove positive self-reported trends in general quality of life (QoL) metrics, statistically significant between-group differences in formal, core mental health parameters remained limited and non-significant (*p* > 0.05).

Holistic and Multi-disciplinary Programs: The internet-delivered SPIRE rehabilitation framework yielded clinical advancements in global subjective well-being, showing prominent cumulative score improvements across the WHOQOL-BREF and ISCI-QOL matrices [62]. However, secondary behavioral tracking—specifically addressing standard Hospital Anxiety and Depression Scale (HADS) thresholds and formal sleep quality parameters—failed to establish statistically significant changes between the active and control configurations [62].

Exercise Intensity Modalities and Enjoyment: Training protocol designs significantly altered patient-reported engagement metrics. Sprint Interval Training (SIT) was perceived by participants as significantly more enjoyable on the Physical Activity Enjoyment Scale (PACES) when directly contrasted against moderate-intensity continuous pathways [58]. Paradoxically, overall exercise self-efficacy metrics (ESES) presented a minor, non-significant decline across both active training cohorts over time [58].

Technology versus Traditional Protocols: Advanced, technology-assisted loco-motor re-training models did not surpass traditional overground physical therapy on generic health-related quality of life dimensions [20,22]. Long-term tracking via the SF-36 matrix demonstrated that conventional overground therapeutic exercises generated superior psychological outcomes compared to stationary robotic setups [50]. Marginally better, non-significant psychological trajectories were generally observed in subacute individuals presenting with incomplete injury profiles, potentially linked to their higher baseline potential for motor recovery [50].

Symptom-Induced Psychological Distress: Target-specific symptom mitigation programs—including structured home exercise frameworks and localized neuromodulation—exerted a strong positive effect on secondary psychological parameters [53,59]. Successfully decreasing physical pain metrics translated directly into a significant, meaningful reduction in secondary psychological distress and a drop in the perceived unpleasantness associated with chronic condition profiles [53,59].

### 3.15. Pain Outcomes

The therapeutic efficacy of targeted physical therapy interventions on chronic neurogenic, secondary musculoskeletal, and myofascial pain presentation was investigated across 4 studies pooling a cumulative sample of 184 participants [53,59,61,62]. Methodological tools evaluated pain outcomes using the Visual Analogue Scale (VAS), the Short-Form McGill Pain Questionnaire (SF-MPQ), the Numerical Pain Scale (NPS), and the International Spinal Cord Injury Pain Basic Data Set (ISCIPBDS). The compiled evidence indicates that pain alleviation is highly effective but localized, with distinct, statistically significant improvements recorded across 3 out of the 4 evaluated papers.

Targeted Neuromodulation and Electrical Stimulation: Specialized electrotherapeutic modalities demonstrated definitive, statistically significant short-term and medium-term analgesic benefits. Transcutaneous Electrical Nerve Stimulation (TENS) applied either directly to validated acupuncture points or peripheral myofascial trigger points provoked a massive, highly significant drop in clinical pain intensity across both the VAS and SF-MPQ indices [59]. Notably, targeting specific acupuncture points proved statistically superior to myofascial sites, inducing significantly longer-lasting analgesia and broader secondary improvements in patient relaxation profiles [59]. Furthermore, active Transcutaneous Tibial Nerve Stimulation (TTNS) setups demonstrated a distinct, statistically significant reduction in autonomic-related clinical discomfort on the NPS compared to sham stimulation configurations [61].

Multidisciplinary and Home-Based Rehabilitation: Comprehensive activation pathways driven by the internet-based SPIRE cognitive–behavioral program succeeded in driving highly superior, statistically significant long-term pain mitigation compared to standard care pathways [62]. Active participation elevated the physical nociceptive stimulus threshold, effectively lowering both the daily frequency and subjective intensity of painful episodes [62]. Conversely, structured home-based shoulder exercise programs (HEP) led to positive reductions in mechanical shoulder pain intensity and local episode durations, but corresponding clinical tracking markers (such as DASH scores) failed to reach formal between-group statistical significance [53]. Across these diverse modalities, successful pain reduction trends were documented consistently regardless of baseline complete or incomplete injury classification profiles.

## 4. Discussion

### 4.1. Methodological Considerations and Evidence Interpretation

The findings of this systematic review demonstrate that while individual physical rehabilitation modalities show promise in targeting specific functional deficits following spinal cord injury (SCI), the evidence is highly heterogeneous and limits the formulation of definitive, universal clinical guidelines. Given the qualitative nature of this synthesis and the structural diversities across the 23 included trials, broad conclusions labeling physical therapy as universally “highly effective” are methodologically unsubstantiated. Instead, the therapeutic value of these interventions must be interpreted with caution, balancing statistically significant breakthroughs against frequent null findings and substantial methodological constraints.

A major conceptual challenge in synthesizing this evidence base arises from aggregating vastly disparate protocols under the broad term “physiotherapy.” Modalities evaluated in this review—ranging from high-technology robotic gait training and Functional Electrical Stimulation (FES) to respiratory muscle training (RMT), Transcutaneous Electrical Nerve Stimulation (TENS), virtual reality (VR) environments, hydrotherapy, and conventional therapeutic exercise—rely on completely distinct physiological mechanisms and target entirely different clinical outcomes. Consequently, these interventions cannot be interpreted as a single, homogeneous treatment category.

Furthermore, the generalizability of these findings is constrained by a distinct recruitment bias within the primary literature. The included trials predominantly evaluate younger cohorts with traumatic spinal cord injuries (tSCIs), leaving non-traumatic spinal cord injury (ntSCI) populations significantly underrepresented. Clinically, while tSCI and ntSCI populations share chronic secondary complications such as motor paralysis, localized muscle atrophy, and spasticity, their baseline profiles differ substantially. Individuals with ntSCI are frequently older and present with progressive ischemic, neoplastic, or degenerative co-morbidities. These factors alter their physiological tolerance to intensive physical conditioning, meaning that high-intensity robotic or aggressive locomotor guidelines derived from tSCI samples cannot be directly extrapolated to non-traumatic cohorts without careful titration.

### 4.2. Muscle Strength

The strength and consistency of evidence regarding muscle strength conditioning is moderate but inconsistent. Out of twelve clinical trials evaluating this domain, only four achieved statistically significant improvements in lower or upper limb muscle torque relative to control cohorts [22,23,47,54]. The remaining eight studies reported statistically equivalent intra-group or between-group changes, indicating that strength gains are not universally sustained across diverse physiotherapeutic protocols. Where improvements were verified, the primary mechanisms driven by targeted neuromuscular activation—such as paraspinal electrical stimulation [21], varied-surface overground walking [47], and biofeedback-assisted exoskeleton training [50]—involved muscle hypertrophy, enhanced motor unit recruitment, and improved synchronization of residual pathways.

Notably, the expression of these structural adaptations was moderated by the baseline neurological status of the participants. Statistically significant adaptations and voluntary strength gains were predominantly observed within subacute cohorts with residual descending neural pathways, providing a viable macro-anatomical substrate for activity-dependent neuroplasticity. Crucially, objective neuro-electrical modifications—such as surface electromyographic (sEMG) amplitude variations—were not restricted to ambulatory cohorts but were successfully triggered within subacute cohorts spanning ASIA B, C, and D profiles, where targeted biofeedback can optimize motor unit recruitment within partially spared or subclinical descending pathways. Conversely, across neurologically complete profiles (ASIA A), voluntary lower-limb muscle recruitment remained structurally blocked. In these complete cohorts, strength-related adaptations were restricted to upper-truncal neurocompensatory mechanisms and accessory respiratory muscle expansions driven by specialized sitting balance or pulmonary interventions rather than direct lower-limb muscle reconditioning.

### 4.3. Gait Ability and Ambulation

The evidence supporting advancements in ambulatory capacity is weak and highly localized. Only two out of seven analyzed studies documented statistically significant improvements in objective spatiotemporal gait parameters [22,47], primarily utilizing intensive exoskeleton-assisted retraining with interactive biofeedback or progressive overground obstacle negotiation. These successful protocols induced short-term expansions in overground walking velocity and optimized stride symmetry.

However, the wider evidence base is characterized by frequent null findings, particularly regarding functional, long-term overground autonomy and patient-reported walking measures, where no statistically significant differences were observed between experimental and control groups. This lack of consistency highlights that high-intensity gait training does not automatically translate into meaningful daily ambulation changes.

The magnitude of loco-motor recovery was completely dependent on the participants’ baseline lesion profile. Progress was almost exclusively restricted to participants with incomplete injuries due to the availability of residual spinal pathways. For individuals presenting with complete or upper-cervical injuries, overground ambulatory retraining did not yield independent walking capabilities, confirming that baseline neurological completeness remains the definitive limiting factor in loco-motor rehabilitation.

### 4.4. Postural Control and Balance

The evidence base for postural stability is relatively robust, with the majority of the reviewed literature (three out of seven studies) reporting statistically significant improvements in trunk control [19,50,51]. The most consistent therapeutic benefits emerged from technology-driven and task-specific frameworks, including immersive VR balance systems [23] and specialized core exercise paradigms combined with concurrent FES [51].

Objectively, these interventions advanced both static and dynamic sitting or standing balance, resulting in an expanded functional base of support, reduced postural sway, and an improved capacity to modulate the body’s center of gravity during self-initiated perturbations.

In contrast to gait and strength outcomes, positive adaptations in postural control were observed across a broader clinical spectrum. Both complete and incomplete SCI participants demonstrated meaningful improvements in sitting stability. This indicates that trunk and core stabilization can be successfully retrained through upper-body neurocompensatory strategies and axial muscle conditioning, even in the presence of complete lower-limb motor deficits. This domain represents one of the most consistent areas of benefit across varying injury severities.

### 4.5. Activities of Daily Living (ADLs) and Autonomy

The strength of evidence concerning functional independence in daily routines is moderate to strong, with three out of four evaluated studies demonstrating statistically significant improvements in standardized ADL metrics, such as the SCIM-III and Modified Barthel Index scores [50,51,60]. The most pronounced benefits were achieved through targeted exercise combined with concurrent electrical stimulation [51], immersive VR environments [23], and EMG biofeedback integrated with conventional physiotherapy [60].

These parameters translated into enhanced functional autonomy within the patients’ immediate micro-environments. However, a critical analysis of the data reveals that these gains were heavily stratified by the level and completeness of the injury. In subjects presenting with incomplete injuries or paraplegia, the interventions facilitated a distinct shift toward partial or full independence, enabling them to execute self-care and transfer tasks without relying on familial caregivers. Conversely, for participants with complete tetraplegia, the observed statistical improvements reflected minor optimization of residual movements or assistive technology mastery rather than a fundamental shift in independent living status.

### 4.6. Respiratory Function

The evidence supporting pulmonary reconditioning is strong and highly consistent, with five out of six articles reporting statistically significant progress in respiratory parameters [21,22,54,55,56]. The most effective protocols included targeted respiratory muscle training (RMT) utilizing specialized pressure-threshold devices [55,56] and structured robotic exoskeleton walking [21,22].

Collectively, these interventions produced measurable improvements in global pulmonary function, including enhanced respiratory muscle recruitment, improved pulmonary ventilation, optimized alveolar gas exchange, and expanded static lung volumes (such as PImax and PEmax expansions).

From a clinical spectrum perspective, these physiological adaptations were observed consistently across both complete and incomplete SCI cohorts. Because RMT primarily targets accessory respiratory muscles and diaphragm excursion—which are often partially spared or highly responsive to neurocompensatory retraining—respiratory conditioning represents a highly generalizable and reliable intervention domain for both paraplegic and tetraplegic populations.

### 4.7. Cardiovascular Function

The strength of evidence regarding cardiovascular control and autonomic regulation is weak and highly inconsistent. Statistically significant improvements were rare across the reviewed literature, with only a small subset of trials demonstrating measurable modifications in hemodynamic control. While successful single-trial interventions—such as whole-body hybrid FES rowing or intensive ambulatory exoskeleton walking—elicited temporary improvements in physical endurance, resting arterial pressure regulation, and cardiovagal baroreflex sensitivity, the overall trends across the 5 evaluated studies were statistically non-significant.

Autonomic dysregulation and orthostatic hypotension remain profound barriers that frequently impair cardiovascular control after SCI, complicating exercise prescription and long-term health management. The available data suggest that while short-term exercise protocols can provoke transient metabolic and central hemodynamic adaptations, they are generally insufficient to systematically restructure or cure underlying autonomic cardiovascular deficits, regardless of whether the injury is classified as complete or incomplete.

### 4.8. Mental Health

The evidence concerning the impact of physical retraining on psychological outcomes is entirely negative. Physiotherapeutic interventions alone do not drive systematic improvements in formal psychiatric metrics, as zero studies reported statistically significant between-group differences on standard anxiety, depression, or health-related quality of life scales (such as the HADS or SF-36 matrices).

This universal lack of statistical significance can be explained by several methodological factors:

*Outcome Hierarchies:* Across all included trials, psychological metrics were systematically treated as secondary or exploratory outcomes, meaning the studies were not statistically powered to detect subtle shifts in mental well-being.

*Intervention Characteristics:* The rehabilitation protocols were exclusively mechanical, physical, and short-term, lacking integrated neuropsychological support components.

Assessment Limitations: Standardized psychometric scales may lack the sensitivity required to capture the complex, long-term emotional adjustments associated with chronic spinal cord injury.

While multidisciplinary frameworks like the SPIRE program [62] noted modest clinical trends toward improved self-reported mood, these changes failed to achieve statistical significance, confirming that mechanical physical therapy cannot substitute for dedicated psychological care.

### 4.9. Pain Assessment

The strength of evidence regarding pain mitigation is weak and highly variable. The therapeutic efficacy of physical interventions on chronic pain is not universally substantiated by the pooled data, with distinct improvements recorded in three out of four evaluated studies [59,61,62]. The specific protocols capable of significantly alleviating discomfort were the comprehensive SPIRE rehabilitation program [62] and targeted Transcutaneous Tibial Nerve Stimulation (TTNS). These interventions triggered neuro-compensatory and neuromodulatory changes that elevated the nociceptive stimulus threshold, decreasing both the clinical frequency and subjective intensity of painful episodes.

However, the remaining half of the literature reported non-significant changes in pain interference scores (such as the DASH or PESS indices). This variability indicates that while localized electrotherapeutic or structured home exercise frameworks may provide transient musculoskeletal relief, they are highly inconsistent when dealing with severe, centralized neuropathic pain, which remains a profound barrier to long-term community reintegration across both complete and incomplete lesion profiles.

### 4.10. Limitations of the Review Process and the Evidence Base

The conclusions of this systematic review must be interpreted in light of several significant limitations, operating at both the review level and the primary evidence level.

#### 4.10.1. Review-Level Limitations

Database and Language Restrictions: The literature search was restricted to studies published in English and confined to three major electronic databases (PubMed, PEDro, and Scopus). The grey literature, conference proceedings, book chapters, and ongoing unpublished clinical trials were not retrieved, introducing potential publication bias.

Methodological Omissions: The search strategy reporting was partially incomplete, and no quantitative meta-analysis could be safely executed due to the compounding diversity of the data. Furthermore, no formal certainty-of-evidence assessment (such as the GRADE framework) was conducted.

Selection Bias and Quality Assessment Limitations: This review excluded studies with a PEDro score below 6/10 based on our pre-registered protocol. While this approach ensured that the included evidence maintained moderate-to-high quality, using a quality score as an eligibility criterion is restrictive. This decision may have excluded potentially informative findings, thereby influencing the comprehensiveness of the evidence synthesis. Furthermore, although the PEDro scale is highly appropriate for the physiotherapy literature, it relies on a cumulative score and does not replace a comprehensive, domain-based risk-of-bias assessment, which represents an additional methodological limitation of this review.

#### 4.10.2. Evidence-Level Limitations

High Clinical Heterogeneity: The included primary trials exhibited extreme diversity regarding baseline population characteristics (mixing traumatic and non-traumatic etiologies), injury levels (tetraplegia versus paraplegia), lesion completeness (ASIA A through D), and injury chronicity (acute, subacute, and chronic phases).

Crucially, a granular limitation within this reporting infrastructure involves the baseline documentation of injury etiology in mixed-cohort trials. While several studies explicitly quantified non-traumatic spinal cord injury (ntSCI) participants within their overall samples, they did not always provide a distinct, disaggregated breakdown between the intervention and control arms. Consequently, pooled baseline syntheses (such as Table 5) must rely on proportional distributions for these specific subgroups. Future primary trials should ensure distinct demographic and clinical reporting for tSCI and ntSCI sub-cohorts to allow for more robust, etiology-specific subgroup analyses in systematic reviews.

Protocol Dissimilarity: There was a total lack of standardization regarding intervention types, treatment dosages, session frequencies. Overall protocol durations exhibited substantial variance, ranging primarily from 2 weeks to 6 months, with a singular long-term pragmatic tracking trial extending up to 20 months.

Variable Timelines and Outcome Measures: Follow-up assessment timelines varied substantially, and studies utilized over 40 different clinical testing batteries to measure overlapping functional domains.

Methodological Weaknesses in Primary Trials: Many included trials exhibiting intrinsic methodological vulnerabilities, most notably a pervasive lack of participant and therapist blinding (an inherent constraint in active physical rehabilitation), unclear or unreported allocation concealment methods, variable follow-up retention rates, and a frequent absence of formal intention-to-treat (ITT) analyses.

## 5. Conclusions

The synthesized literature indicates that physical therapy interventions post-spinal cord injury should not be broadly characterized as uniformly effective across all functional domains. Instead, their therapeutic impact is highly domain-specific, localized, and moderated by the baseline neurological characteristics of the patient population.

The qualitative synthesis reveals that the most consistent and robust evidence of benefit resides in respiratory muscle training and technology-driven postural control interventions, which demonstrate a capacity to advance pulmonary parameters and trunk stability across a wide clinical spectrum, including both complete and incomplete lesions. Conversely, the evidence supporting improvements in muscle strength, activities of daily living, and overground gait parameters is moderate to weak, with positive outcomes largely restricted to motor-incomplete (ASIA C and D) cohorts where residual descending neural pathways are preserved. Furthermore, the compiled data indicate that isolated physical interventions do not drive systematic improvements in formal mental health metrics and yield highly inconsistent results regarding chronic pain mitigation and cardiovascular autonomic regulation.

Given the extreme clinical and methodological heterogeneity in intervention modalities, treatment dosages, and follow-up timelines across the evaluated trials, the current evidence is insufficient to establish uniform, structural clinical guidelines. Future research requires large-scale, multi-center randomized controlled trials utilizing standardized protocols, definitive follow-up intervals, and rigorous evidence grading to fully isolate the long-term efficacy of specific rehabilitative frameworks in both traumatic and underrepresented non-traumatic spinal cord injury cohorts.

## Figures and Tables

**Table 1:** Study Characteristics.

Article	Country/Year	Injury Type/Pathology	Sample Size (N)
Zwijgers E et al. [46]	2024	Incomplete ASIA C–D	EG: 17, CG: 18
Goel T et al. [23]	2023	Incomplete ASIA B–D	EG1: 9, EG2: 9
Amatachaya S et al. [47]	2021	Incomplete ASIA C–D	EG: 28, CG: 26
Guo Y et al. [24]	2021	Incomplete ASIA B (T10–L1)	EG: 18, CG: 17
Solinsky R et al. [48]	2021	Incomplete/Complete ASIA A–C (C1–T10)	EG1: 16, EG2: 11, CG: 11
Xiang XN et al. [19]	2021	Incomplete/Complete ASIA A–C (T3–L2)	EG: 9, CG: 9
Lotter JK et al. [49]	2020	Incomplete ASIA C (T10 or higher)	EG: 9, CG: 8
Piira A et al. [50]	2020	Incomplete ASIA C–D	EG: 16, CG: 21
Rahimi M et al. [51]	2020	Complete Paraplegia ASIA A-B (T2–T13)	EG1: 5, EG2: 5, CG: 7
Chen LW et al. [52]	2020	Incomplete/Complete ASIA A–D	EG: 60, CG: 60
Cardenas DD et al. [53]	2020	Incomplete/Complete ASIA A–D	EG: 17, CG: 15
Jo HJ et al. [54]	2020	Incomplete/Complete ASIA A–D (C2–L3)	EG1: 13, EG2: 13, CG: 12
Vivodtzev I et al. [55]	2020	Incomplete/Complete ASIA A–C (C4–T8)	EG: 10, CG: 10
Boswell-Ruys CL et al. [56]	2020	Incomplete/Complete ASIA A–C (C4–C8)	EG: 30, CG: 32
Soumyashree S et al. [57]	2020	Incomplete/Complete ASIA A–D (T1–T12)	EG: 15, CG: 15
Mcleod JC et al. [58]	2020	Incomplete/Complete ASIA A–D (Below C2)	EG1: 10, EG2: 10
Chiou YF et al. [59]	2020	Incomplete/Complete ASIA A–D	EG1: 32, EG2: 32
Cheung EYY et al. [20]	2019	Incomplete ASIA B (L5 or higher)	EG: 8, CG: 8
Gorman PH et al. [21]	2019	Incomplete ASIA C–D (CMISCI C2–T12)	EG1: 20, EG2: 17
Amatachaya S et al. [60]	2019	Incomplete ASIA C-D	EG1: 11, EG2: 11
Holman ME et al. [22]	2019	Complete ASIA A–B (C5–L2)	EG: 11, CG: 11
Stampas A et al. [61]	2019	Incomplete/Complete ASIA A–D (Up to T9)	EG: 12, CG: 7
Burke D et al. [62]	2019	Incomplete/Complete ASIA A–D	EG: 35, CG: 34

Abbreviations: EG, Experimental Group; CG, Control Group; ASIA, American Spinal Injury Association Impairment Scale.

**Table 2:** Intervention Details.

	Experimental Intervention Target (EG)	Control Intervention Target (CG)
Zwijgers E et al., 2024 [46]	Walking Adaptability Training	Conventional Locomotor & Strength Training
Goel T et al., 2023 [23]	EG1: Virtual Reality (VR) + Conventional PT; EG2: Functional Electrical Stimulation (FES) + Conventional PT	No standalone conventional control group
Amatachaya S et al., 2021 [47]	Walking training on a walking track with visual cues (WTDC)	Overground walking training on standard flat surfaces
Guo Y et al., 2021 [24]	EMG Biofeedback + Conventional Physical Therapy	Conventional Physical Therapy alone
Solinsky R et al., 2021 [48]	EG1: Whole-body exercise (hybrid FES rowing); EG2: Arms-only rowing	No structured exercise protocol
Xiang XN et al., 2021 [19]	Exoskeleton-assisted walking training (AIDER)	Strength training + aerobic exercise + dynamic balance training
Lotter JK et al., 2020 [49]	Task-Specific Loco-motor Training	Impairment-based training protocols
Piira A et al., 2020 [50]	Body-Weight-Supported Treadmill Training (BWSTT)	Conventional Physical Therapy
Rahimi M et al., 2020 [51]	EG1: Advanced Mat Exercises EG2: Advanced Mat Exercises + Functional Electrical Stimulation (FES)	Conventional Physical Therapy
Chen LW et al., 2020 [52]	Physiotherapy + 10,000 voluntary muscle contractions	Conventional Physiotherapy protocols alone
Cardenas DD et al., 2020 [53]	Structured Home Exercise Program (HEP)	Education-only control condition
Jo HJ et al., 2020 [54]	EG1: Paired Corticospinal-Motor Neuronal Stimulation (PCMS) + Exercise EG2: PCMS training alone	Sham PCMS protocols + Physical Exercise
Vivodtzev I et al., 2020 [55]	FES Rowing + Non-Invasive Mechanical Ventilation (NIV)	Sham FES protocols + Non-Invasive Ventilation (NIV)
Boswell-Ruys CL et al., 2020 [56]	Active Respiratory Muscle Training (RMT) utilizing resistive devices	Sham Respiratory Muscle Training (RMT)
Soumyashree S et al., 2020 [57]	Threshold Inspiratory Muscle Training (IMT)	Conventional respiratory physiotherapy protocols
Mcleod JC et al., 2020 [58]	EG1: Moderate-Intensity Continuous Training (MICT) EG2: Sprint Interval Training (SIT)	Comparison between active intervention groups
Chiou YF et al., 2020 [59]	EG1: TENS applied on validated acupuncture points EG2: TENS applied directly on myofascial trigger points	Comparison between two alternative TENS modalities
Cheung EYY et al., 2019 [20]	Robotic-assisted body-weight-supported treadmill training + EMG-Biofeedback	Passive lower limb mobilization training
Gorman PH et al., 2019 [21]	EG1: Powered Robotic Exoskeleton Therapy (RT)	EG2/CG: Aquatic Therapy (AT) protocols
Amatachaya S et al., 2019 [60]	EG1: Dual-task obstacle crossing training (DTOC)	EG2/CG: Single-task obstacle crossing training
Holman ME et al., 2019 [22]	Testosterone Replacement + Progressive Resistance Training (TRT + RT)	Testosterone replacement therapy alone
Stampas A et al., 2019 [61]	Transcutaneous Tibial Nerve Stimulation (TTNS)	Sham Transcutaneous Tibial Nerve Stimulation (TTNS)
Burke D et al., 2019 [62]	SPIRE cognitive-behavioral and physical activation program	Conventional Physiotherapy regimens

Individual protocols tracking intervention dosage and longitudinal parameters varied significantly, ranging across a multi-center duration span of 1.5 to 20 months.

**Table 3:** Outcome Measures and Initial Targets.

Article	Tests & Assessment Scales	Key Results & Direction of Effect
Zwijgers E et al., 2024 [46]	2mWT, SCI-FAP, ABC scale	Significant improvement in walking capacity, functional gait, balance confidence, and participation in the EG. No significant difference observed regarding walking speed. Significant improvement in exercise execution time across both groups.
Goel T et al., 2023 [23]	MFR, FIST, SCIM-III	Both VR training and FES were effective in improving sitting balance in individuals with paraplegia. VR demonstrated overall statistical superiority over FES.
Amatachaya S et al., 2021 [47]	10MWT, TUG, FTSST, 6MWDT	EG demonstrated significant improvement in functional capacity (10MWT, TUG, FTSST, and 6MWT) following 2 and 4 weeks of training. Significantly lower incidence of falls recorded in the EG.
Guo Y et al., 2021 [24]	MMT, Modified Ashworth Scale, MBI, sEMG, quadriceps strength (RMS)	Significant improvement in quadriceps sEMG values in the EG. Muscle strength and ADLs increased across both groups, with no major between-group differences.
Solinsky R et al., 2021 [48]	VO2max, Resting Systolic/Diastolic BP	Individuals with paraplegia demonstrated a significantly greater increase in cardiovagal baroreflex sensitivity compared to those with tetraplegia. Whole-body hybrid FES rowing demonstrates significant improvements in BP regulation.

Abbreviations: 2mWT: 2-Minute Walk Test; 10MWT: 10-Meter Walk Test; 6MWDT: 6-Minute Wheelchair Distance Test; ABC scale: Activities-specific Balance Confidence scale; ADLs: Activities of Daily Living; BP: Blood Pressure; EG: Experimental Group; FES: Functional Electrical Stimulation; FIST: Function In Sitting Test; FTSST: Five-Times-Sit-to-Stand Test; MBI: Modified Barthel Index; MFR: Functional Reach Test (Modified); MMT: Manual Muscle Testing; RMS: Root Mean Square (for EMG signal amplitude); SCI-FAP: Spinal Cord Injury Functional Ambulation Profile; sEMG: Surface Electromyographic activity/Surface Electromyography; TUG: Timed Up and Go; VO2max: Maximal Oxygen Consumption; VR: Virtual Reality.

**Table 4:** Main Findings, Timelines, and Significance Trends Across Included Trials.

Article	Synthesis of Main Findings, Direction & Observed Significance	Follow-Up Timeline & Classification
Zwijgers E et al., 2024 [46]	Significant improvement in walking capacity, functional gait, balance confidence, and participation in the EG. No significant differences in overground gait speed.	1.5 to 20 Months (Variable Long-term)
Goel T et al., 2023 [23]	Both VR and FES were effective in improving sitting balance. VR demonstrated overall statistical superiority. Significant SCIM-III gains.	4 Weeks (Short-term)
Amatachaya S et al., 2021 [47]	Significant improvement in functional capacity (10MWT, TUG, FTSST, 6MWT) and a lower incidence of falls in the EG.	2 & 4 Weeks (Short-term)
Guo Y et al., 2021 [24]	Significant improvement in quadriceps sEMG values in the EG. Muscle strength and ADLs increased equivalently across both groups.	4 Weeks (Short-term/Post-intervention)
Solinsky R et al., 2021 [48]	Paraplegic participants showed a significantly greater increase in cardiovagal baroreflex sensitivity than tetraplegic participants. Hybrid FES rowing significantly improved BP regulation.	6 Weeks (Medium-term/Post-intervention)
Xiang XN et al., 2021 [19]	EG showed raw improvements in walking distance and pulmonary parameters, but changes did not reach statistical significance (*p* > 0.05). Minimal LEMS changes.	8 Weeks (Medium-term/Post-intervention)
Lotter JK et al., 2020 [49]	Task-Specific training was significantly superior for peak treadmill speed, gait, and motor outcomes. Impairment training improved recumbent stepping power.	12 Weeks (Medium-term/Post-intervention)
Piira A et al., 2020 [50]	Significant improvements noted in LEMS, EBSE, psychological parameters, and baseline expectation fulfillment following BWSLT.	8 Weeks (Medium-term/Post-intervention)
Rahimi M et al., 2020 [51]	Significant improvements across 4 SCIM-III transfer items in both EGs, alongside raw progress in endurance, upper limb strength, and core stability.	4 Weeks (Short-term/Post-intervention)
Chen LW et al., 2020 [52]	Significantly greater improvement in muscle power in the EG. Minimal objective effect on voluntary strength, but EG perceived greater progress.	8 Weeks (Medium-term)
Cardenas DD et al., 2020 [53]	Significant reduction in shoulder pain intensity and episode duration for the HEP group. PESS and DASH scores improved non-significantly across both groups.	6 Months (Medium-term/Post-intervention)
Jo HJ et al., 2020 [54]	Corticospinal response amplitudes (TMS) and MVC magnitudes significantly increased in active groups but not sham. Improved walking capacity in incomplete injuries.	2 Weeks (Short-term/Post-intervention)
Vivodtzev I et al., 2020 [55]	FES + NIV group significantly altered respiratory patterns and gas exchange. Incomplete injury profiles (ASIA C) trended toward greater VO2peak gains.	6 Weeks (Medium-term/Post-intervention)
Boswell-Ruys CL et al., 2020 [56]	PImax was significantly higher in active RMT for all lesion profiles. Non-significant expiratory strength changes; sham group had significantly more complications.	8 Weeks (Medium-term/Post-intervention)
Soumyashree S et al., 2020 [57]	EG scored significantly higher on 12MWAT, MSFT, 6MPT, MIP, and MEP (*p* < 0.05), with significantly reduced dyspnea scores.	4 Weeks (Short-term/Post-intervention)
Mcleod JC et al., 2020 [58]	SIT group elicited significantly higher cardiovascular responses and RPE than MICT. Peak power output (PPO) improved equivalently (*p* > 0.05).	12 Weeks (Medium-term/Post-intervention)
Chiou YF et al., 2020 [59]	TENS at both sites significantly reduced myofascial pain. Acupuncture points were statistically superior with longer-lasting analgesia. Anxiety/depression fell equally (*p* > 0.05).	4 Weeks (Short-term/Post-intervention)
Cheung EYY et al., 2019 [20]	Significant improvement for the EG in WISCI-II, SCIM-III, and peak oxygen consumption. Significant increase in PEF and overground gait control.	12 Weeks (Medium-term/Post-intervention)
Gorman PH et al., 2019 [21]	Both groups safely enhanced cardiorespiratory metrics. RT exerted a significantly more positive effect on VO2peak responses compared to AT.	16 Weeks (Medium-term/Post-intervention)
Amatachaya S et al., 2019 [60]	Both groups exhibited significant improvements in functional tests. The error rate decreased significantly specifically in the DTOC group.	4 Weeks (Short-term/Post-intervention)
Holman ME et al., 2019 [22]	TRT + RT group showed significant improvements in muscle quality, torque, size, and contractile properties. Equal lower limb lean mass increases across both groups.	6 Weeks (Medium-term/Post-intervention)
Stampas A et al., 2019 [61]	Bladder capacity and DSD episodes remained stable in the TTNS group but worsened significantly in the sham group. Active TTNS was effective for great toe flexion.	2 Weeks (Short-term/Post-intervention)
Burke D et al., 2019 [62]	Pain levels improved significantly more in the EG. No statistically significant between-group differences were observed in overall quality of life or HADS metrics.	6 Months (Medium-term/Post-intervention)

Abbreviations: 10MWT: 10-Meter Walk Test; 12MWAT: 12-Minute Wheelchair Aerobic Test; 2mWT: 2-Minute Walk Test; 6MPT: 6-Minute Propulsion Test; 6MWT: 6-Minute Walk Test; ADLs: Activities of Daily Living; ASIA: American Spinal Injury Association standard neurological classification; AT: Aerobic Training; BP: Blood Pressure; BWSLT: Body-Weight-Supported Treadmill Training; DASH: Disabilities of the Arm, Shoulder and Hand questionnaire; DSD: Detrusor Sphincter Dyssynergia; DTOC: Dual-Task Overground Condition; EBSE: Evaluation of Balance for Spinal Cord Injury; EG: Experimental Group; FES: Functional Electrical Stimulation; FTSST: Five-Times-Sit-to-Stand Test; HADS: Hospital Anxiety and Depression Scale; HEP: Home Exercise Program; LEMS: Lower Extremity Motor Score; MEP: Maximal Expiratory Pressure; MICT: Moderate-Intensity Continuous Training; MIP/PImax: Maximal Inspiratory Pressure; MVC: Maximal Voluntary Contraction; NIV: Non-Invasive Ventilation; PEF: Peak Expiratory Flow; PESS: Wheelchair User’s Shoulder Pain Evaluation; PPO: Peak Power Output; RMT: Respiratory Muscle Training; RPE: Rating of Perceived Exertion; RT: Resistance Training; SCIM-III: Spinal Cord Injury Independence Measure (Version III); sEMG: Surface Electromyography; SIT: Sprint Interval Training; TENS: Transcutaneous Electrical Nerve Stimulation; TMS: Transcranial Magnetic Stimulation; TRT: Neuromuscular Electrical Stimulation-Evoked Resistance Training; TTNS: Transcutaneous Tibial Nerve Stimulation; TUG: Timed Up and Go; VO2peak: Peak Oxygen Consumption; VR: Virtual Reality; WISCI-II: Walking Index for Spinal Cord Injury (Version II).

**Table 5:** Baseline demographic and clinical characteristics of participants.

Characteristic	Intervention Group (EG)	Control Group (CG)	Total (N)
Mean Age (years)	42.9	41.5	42.2
Gender, *n*			
Male participants	293	215	508
Female participants	89	89	178
Injury Level, *n*			
Paraplegic participants	161	154	315
Tetraplegic participants	220	198	418
Injury Etiology, *n* ^1^			
Traumatic SCI (tSCI)	274	272	546
Non-Traumatic SCI (ntSCI)	55	46	101
Neurological Completeness, *n*			
Incomplete SCI	218	197	415
Complete SCI	159	153	312

^1^ Abbreviations: EG, Experimental Group; CG, Control Group; SCI, Spinal Cord Injury. Cumulative values for injury etiology represent the reporting cohorts (*n* = 546 tSCI and *n* = 101 ntSCI cases) extracted across the 23 included trials. For studies where etiology was not segregated by group at baseline, cases were distributed proportionally.

**Table 6:** Methodological Quality of Included Studies (PEDro Scale).

Study	Q1	Q2	Q3	Q4	Q5	Q6	Q7	Q8	Q9	Q10	Q11	Total Score
Boswell-Ruys CL et al., 2020 [56]	Y	1	1	1	1	1	1	1	1	1	1	10/10
Vivodtzev I et al., 2020 [55]	Y	1	1	1	1	0	1	1	1	1	1	9/10
Stampas A et al., 2019 [61]	Y	1	1	1	1	0	1	1	1	1	1	9/10
Xiang XN et al., 2021 [19]	Y	1	0	1	1	0	1	1	1	1	1	8/10
Chen LW et al., 2020 [52]	Y	1	1	1	0	0	1	1	1	1	1	8/10
Rahimi M et al., 2020 [51]	Y	1	0	1	1	0	1	1	1	1	1	8/10
Chiou YF et al., 2020 [59]	Y	1	1	1	0	0	1	1	1	1	1	8/10
Cheung EYY et al., 2019 [20]	Y	1	0	1	1	0	1	1	1	1	1	8/10
Zwijgers E et al., 2024 [46]	Y	1	0	1	0	0	1	1	1	1	1	7/10
Piira A et al., 2020 [50]	Y	1	0	1	0	0	1	1	1	1	1	7/10
Soumyashree S et al., 2020 [57]	Y	1	0	1	0	0	1	1	1	1	1	7/10
Burke D et al., 2019 [62]	Y	1	0	1	0	0	1	1	1	1	1	7/10
Holman ME et al., 2019 [22]	Y	1	0	1	0	0	1	1	1	1	1	7/10
Gorman PH et al., 2019 [21]	Y	1	0	1	0	0	1	1	1	1	1	7/10
Amatachaya S et al., 2019 [60]	Y	1	0	1	0	0	1	1	1	1	1	7/10
Goel T et al., 2023 [23]	Y	1	0	1	0	0	0	1	1	1	1	6/10
Amatachaya S et al., 2021 [47]	Y	1	0	1	0	0	0	1	1	1	1	6/10
Solinsky R et al., 2021 [48]	Y	1	0	1	0	0	0	1	1	1	1	6/10
Guo Y et al., 2021 [24]	Y	1	0	1	0	0	0	1	1	1	1	6/10
Lotter JK et al., 2020 [49]	Y	1	0	1	0	0	0	1	1	1	1	6/10
Cardenas DD et al., 2020 [53]	Y	1	0	1	0	0	0	1	1	1	1	6/10
Jo HJ et al., 2020 [54]	Y	1	0	1	0	0	0	1	1	1	1	6/10
Mcleod JC et al., 2020 [58]	Y	1	0	1	0	0	0	1	1	1	1	6/10

Note: Q1 (Eligibility criteria) is used to confirm external validity and is not included in the total PEDro score calculation. Q2: Random allocation; Q3: Concealed allocation; Q4: Baseline similarity; Q5: Blinding of subjects; Q6: Blinding of therapists; Q7: Blinding of assessors; Q8: Adequate follow-up (>85%); Q9: Intention-to-treat analysis; Q10: Between-group statistical comparisons; Q11: Point measures and variability.

**Table 7:** Clinical Domains and Assessment Instruments/Metrics.

Clinical Domain	Assessment Instruments/Metrics
Muscular Strength	SP, FTSST, MVC, PT, PPO, LEMS
Gait Ability	10MWT, WISCI, TUG, 2mWT, SCI-FAP, PROMIS
Balance	MFR, BBS, ABC, FIST, QUR, T-K, FTSST, TUG
Activities of Daily Living (ADLs)	SCIM, MBI
Respiratory Function	MBS, FEV_1_, FVC, FEF_25/50/75_, PEF, OUES, SGRQ, TLC, PImax, PEmax
Cardiovascular Function	6MWT, VO_2_peak, RSBP, RDBP, HR, 12MWAT, SpO_2_, MSFT, 6MPT
Mental Health & Quality of Life	HADS, PACES, WHOQOL-BREF, ISCI-QOL, SF-36, ESES, AU
Pain Assessment	VAS, EQ-5D VAS, SF-MPQ, ISCIPBDS, NPS

## Data Availability

The systematic review protocol is publicly accessible on the Open Science Framework (OSF) platform (https://osf.io/v64m2, accessed on 13 May 2026). Template data extraction forms and all other materials used during the narrative synthesis are not publicly hosted but are available from the corresponding author upon reasonable request.

## Supplementary Materials

The following supporting information can be downloaded at: https://www.mdpi.com/article/10.3390/healthcare14142048/s1, Table S1: Categorical matrix illustrating the structural distribution and therapeutic efficacy of the included clinical trials (N = 23) across primary functional evaluation domains; Table S2: Demographic and clinical stratification matrix mapping the baseline characteristics of the total pooled participant sample (N = 730).

## Abbreviations

The following abbreviations are used in this manuscript:

Abbreviation	Full Term
10MWT	10-Meter Walk Test
2mWT	2-Minute Walk Test
6MPT	6-Minute Propulsion Test
6MWT	6-Minute Walk Test
12MWAT	12-Minute Wheelchair Aerobic Test
ABC	Activity-specific Balance Confidence scale
AU	Muscle Activity Units
BBS	Berg Balance Scale
BDI	Beck Depression Inventory
BP	Blood Pressure
BPI	Brief Pain Inventory
BWSLT/BWSTT	Body-Weight-Supported Treadmill Training
CG	Control Group
EG	Experimental/Intervention Group
EQ-5D VAS	EuroQol 5-Dimension Visual Analogue Scale
ESES	Exercise Self-Efficacy Scale
FEF	Forced Expiratory Flow
FEV_1_	Forced Expiratory Volume in 1 s
FIST	Function In Sitting Test
FTSST	Five-Times-Sit-to-Stand Test
FVC	Forced Vital Capacity
HADS	Hospital Anxiety and Depression Scale
HR	Heart Rate
ISCIPBDS	International Spinal Cord Injury Pain Basic Data Set
ISCI-QOL	International Spinal Cord Injury Quality of Life Basic Data Set
LEMS	Lower Extremity Motor Score
MBI	Modified Barthel Index
MBS	Modified Borg Scale
MFR	Modified Functional Reach
MIP/MEP	Maximal Inspiratory/Expiratory Pressure
MMT	Manual Muscle Testing
MSFT	Multi-Stage Fitness Test
MVC	Maximum Voluntary Contraction
MVV	Maximal Voluntary Ventilation
NPS	Numerical Pain Scale
OUES	Oxygen Uptake Efficiency Slope
PACES	Physical Activity Enjoyment Scale
PEF	Peak Expiratory Flow
PEmax	Maximal Expiratory Pressure
PImax	Maximal Inspiratory Pressure
PPO	Peak Power Output
PROMIS	Patient-Reported Outcomes Measurement Information System
PSFS	Patient-Specific Functional Scale
PSQI	Pittsburgh Sleep Quality Index
PT	Peak Torque
QUR	Quadruped Reaching
RDBP	Resting Diastolic Blood Pressure
RMS	Root Mean Square
RSBP	Resting Systolic Blood Pressure
SCI-FAP	Spinal Cord Injury Functional Ambulation Profile
SCIM	Spinal Cord Independence Measure
sEMG	Surface Electromyography
SF-36	36-Item Short Form Survey
SF-MPQ	Short-Form McGill Pain Questionnaire
SGRQ	St. George’s Respiratory Questionnaire
SP	Stepping Power
SpO_2_	Peripheral Oxygen Saturation
T-K	Tall-Kneeling
TLC	Total Lung Capacity
TMS	Transcranial Magnetic Stimulation
TUG	Timed Up and Go
UEMS	Upper Extremity Motor Score
VAS	Visual Analogue Scale
VO_2_peak/VO_2_max	Peak/Maximal Oxygen Consumption
WHOQOL-BREF	World Health Organization Quality of Life Brief Version
WISCI	Walking Index for Spinal Cord Injury
