# Supplementary material for: Physical Therapy for Traumatic and Non-Traumatic Spinal Cord Injuries in Adults: A Systematic Review
Source: Healthcare (Basel). 2026 Jul 8;14(14):2048. doi: 10.3390/healthcare14142048 (PMC13409793; doi:10.3390/healthcare14142048)
Supplement: Supplementary file 1 [file healthcare-14-02048-s001.zip › healthcare-4352185-supplementary.pdf]

## Supplementary Materials

**Table S1.** Categorical matrix illustrating the structural distribution and therapeutic efficacy of the included clinical trials (N = 23) across primary functional evaluation domains.

| Functional Evaluation Domain | Total Trials Evaluated (N=23) | Statistically Significant Improvement ($p < 0.05$) | Null / Equivalent Outcomes ($p >= 0.05$) | Primary Modalities with Highest Efficacy |
|---|---|---|---|---|
| **Muscle Strength & Metrics** | 12 | 4 | 8 | Paraspinal NMES + Exercise, Exoskeleton + EMG biofeedback |
| **Gait & Overground Ambulation** | 14 | 6 | 8 | Robotic-assisted locomotor training, Unstable surface walking |
| **Postural Control & Balance** | 9 | 5 | 4 | Specialized sitting balance protocols, Core stabilization training |
| **Activities of Daily Living (ADLs)** | 11 | 3 | 8 | Task-specific functional resistance training, Hydrotherapy |
| **Cardiorespiratory & Endurance** | 7 | 4 | 3 | Respiratory Muscle Training (RMT), Arm |

| | | | | |
|---|---|---|---|---|
| | | | | crank ergometry |
| **Quality of Life & Pain Assessment** | 8 | 3 | 5 | Integrated hydrotherapy, Neuromuscular electrical stimulation |

**Table S2.** Demographic and clinical stratification matrix mapping the baseline characteristics of the total pooled participant sample (N = 730).

| Clinical Parameter | Sub-category Matrix | Participant Volume (N = 730) | Percentage Distribution (%) |
|---|---|---|---|
| **Injury Etiology** | Traumatic Spinal Cord Injury (tSCI) | 511 | 70% |
| **Injury Etiology** | Non-Traumatic Spinal Cord Injury (ntSCI) | 219 | 30% |
| **Neurological Level** | Cervical (Tetraplegia) | 343 | 47% |
| **Neurological Level** | Thoracic / Lumbar (Paraplegia) | 285 | 39% |
| **Neurological Level** | Not Reported / Unspecified | 102 | 14% |
| **Injury Completeness** | Motor Incomplete (AIS C or D) | 460 | 63% |
| **Injury Completeness** | Motor Complete (AIS A or B) | 270 | 37% |

| | | | |
|---|---|---|---|
| **Chronicity Phase** | Subacute (< 6 months post-injury) | 292 | 40% |
| **Chronicity Phase** | Chronic (>= 6 months post-injury) | 438 | 60% |
